# An Improved Temporal Fusion Transformers Model for Predicting Supply Air Temperature in High-Speed Railway Carriages

**DOI:** 10.3390/e24081111

**Published:** 2022-08-12

**Authors:** Guoce Feng, Lei Zhang, Feifan Ai, Yirui Zhang, Yupeng Hou

**Affiliations:** School of Artificial Intelligence and Data Science, Hebei University of Technology, Tianjin 300401, China

**Keywords:** supply air temperature forecasting, transformer, temporal fusion transformers, causal convolution

## Abstract

A key element for reducing energy consumption and improving thermal comfort on high-speed rail is controlling air-conditioning temperature. Accurate prediction of air supply temperature is aimed at improving control effects. Existing studies of supply air temperature prediction models are interdisciplinary, involving heat transfer science and computer science, where the problem is defined as time-series prediction. However, the model is widely accepted as a complex model that is nonlinear and dynamic. That makes it difficult for existing statistical and deep learning methods, e.g., autoregressive integrated moving average model (ARIMA), convolutional neural network (CNN), and long short-term memory network (LSTM), to fully capture the interaction between these variables and provide accurate prediction results. Recent studies have shown the potential of the Transformer to increase the prediction capacity. This paper offers an improved temporal fusion transformers (TFT) prediction model for supply air temperature in high-speed train carriages to tackle these challenges, with two improvements: (i) Double-convolutional residual encoder structure based on dilated causal convolution; (ii) Spatio-temporal double-gated structure based on Gated Linear Units. Moreover, this study designs a loss function suitable for general long sequence time-series forecast tasks for temperature forecasting. Empirical simulations using a high-speed rail air-conditioning operation dataset at a specific location in China show that the temperature prediction of the two units using the improved TFT model improves the MAPE by 21.70% and 11.73%, respectively the original model. Furthermore, experiments demonstrate that the model effectively outperforms seven popular methods on time series computing tasks, and the attention of the prediction problem in the time dimension is analyzed.

## 1. Introduction

The comfort of taking high-speed rail is receiving a lot of attention from passengers; there has been rapid development of the high-speed rail industry and a sharp increase in the number of people who choose to travel by high-speed rail [1,2,3]. The change in the environment during the high-speed train driving is a critical ingredient that causes the temperature change in the high-speed train compartment, such as outside temperature, light intensity, speed, and other factors [4]. Because of its high hysteresis, the air conditioner’s air supply temperature will fluctuate with the temperature of the high-speed train compartment, causing human discomfort and making control difficult. An accurate estimate of the supply air temperature is critical to improving thermal comfort, reducing the energy required for air conditioning, and building high-speed train air-conditioning management systems. According to the actual forecast demand, it is necessary to predict the value of the supply air temperature for a long time to improve the control effect of the control system. In a complicated environment, it is therefore challenging to obtain accurate, dependable, high-quality, high-precision, and long-term temperature forecasts for high-speed train carriages. Thus, precisely predicting supply air temperature is a challenging and significant task.

Methods for supply air temperature prediction can be summarized into three categories: physical models prediction methods, statistical prediction methods, and artificial intelligence prediction methods.

Physical models predict the supply air temperature by building a heat transfer model. The components of the high-speed rail air-conditioning system are primarily composed of compressors, condenser fans, and other components. The thermodynamic model of the air conditioner has been proposed in some research, but, due to the model’s complexity, only a simplified and conditional model can be supplied [5]. The heat transfer prediction model based on thermodynamics is quite complex, and the parameters of the heat transfer model are different, so the supply air temperature prediction based on the physical model cannot satisfy the demand.

Statistical models for temporal forecasting include autoregressive (AR) [6], auto-regressive integrated moving average (ARIMA) [7], and exponential smoothing (ETS). AR uses the dependent relationship of the historical time series of the prediction target between the values in different periods to establish a regression equation for the prediction. Combining AR and MA approaches results in ARMA, which captures the linear relationship between variables across time and yields prediction results. The ARIMA model evolved from the Auto-Regressive Moving Average (ARMA) model, which differentially processes non-stationary data before modeling with ARMA. In addition to these methods, there are numerous prediction techniques such as gray forecast [8], the Kalman filter [9], and the Hammerstein auto-regressive method [10], among others. Most statistical methods build a time series model using past temperature data that can extract linear features, and they perform well in ultra-short-term or single-step forecasting. However, these methods cannot be employed to anticipate long-term temperatures accurately. Long-term dependencies between variables deteriorate over time as well.

Artificial intelligence forecasting methods are employed for time series forecasting, including traditional machine learning and the more prevalent deep learning methods. In practical applications, machine learning methods mainly include support vector regression (SVR) [11], random forest (RF) [12], and XGBoost [13]. These approaches have experienced quality assurance in many prediction tasks and performed well in some tests. However, standard machine learning algorithms cannot handle data in the time dimension since the relevance of data at each location is identical for these methods, preventing the extraction of usable knowledge in the time dimension. With the rapid development of deep learning, some meaningful learning methods have been improved and applied in the direction more suitable for extracting time series features, such as Long Short-Term Memory Neural Network (LSTM), Temporal Convolutional Network (TCN), and Transformer. The Recurrent Neural Network (RNN) processes preceding and subsequent related input by memorizing features, ideally suited for processing time series. As an enhanced recurrent neural network model, LSTM can successfully extract relevant information from historical data, and its “gate” structure can selectively extract useful historical information [14]. TCN is a network specialized in processing sequence information, which has the high parallel processing capability, stable gradient, and a more flexible receptive field that LSTM lacks [15,16,17]. In addition, with the excellent performance of attention mechanisms in the image and natural language processing, many scholars have also begun to apply attention mechanisms to time series prediction tasks [18,19]. The Transformer model uses the size of the attention weight to identify the salient part of the input for each instance, enhancing the model’s interpretability. The Transformer model far outperforms the RNN model in capturing long-range dependencies.

In the process of high-speed train driving, the environment of the air conditioning system in the carriage is complicated and changing. Prediction of supply air temperature is a very challenging task. For this reason, this study selected the TFT model for the first time in multivariate and interpretable high-performance temperature prediction [20]. TFT models are under various variable input structures, while TFT can provide insightful interpretations of temporal dynamics. Furthermore, a novel design is introduced under its original architecture to enhance the performance of TFT and extract deeper data features. Specifically, our improvements are as follows:

- Double-dilated causal convolutional network (DDN), two causal convolutions with different dilation factor sizes, forms a layer of DDN. A multi-layer DDN structure includes a new known variable encoder component to replace the LSTM encoder structure;
- Double gating residual network (DGRN), a temporal convolution structure, is added to the original gated residual network to minimize the influence of irrelevant variables and variable moments.

In addition, a shape and time distortion loss function (DILATE) based on dynamic time warping is introduced to guide the model in the direction of sequence similarity during training [21]. In practical temperature prediction problems, the closer time nodes in the future are better than those farther away. The effect of time nodes will be pretty poor. In this regard, a time distance loss matrix is added to the loss function so that the model pays greater attention to the learning at longer time distances.

The experiments in this paper are based on the data on the operation of the air-conditioning system of a high-speed railway in Shanghai, China, during summer driving. The framework of this paper is as follows: Section 2 describes the underlying theory, Section 3 describes the model framework and two improvements, Section 4 describes the loss function, Section 5 plots and analyzes experimental results, and Section 6 presents conclusions.

## 2. Methodology

### 2.1. Time Series Forecasting Problem

**Figure 1:**
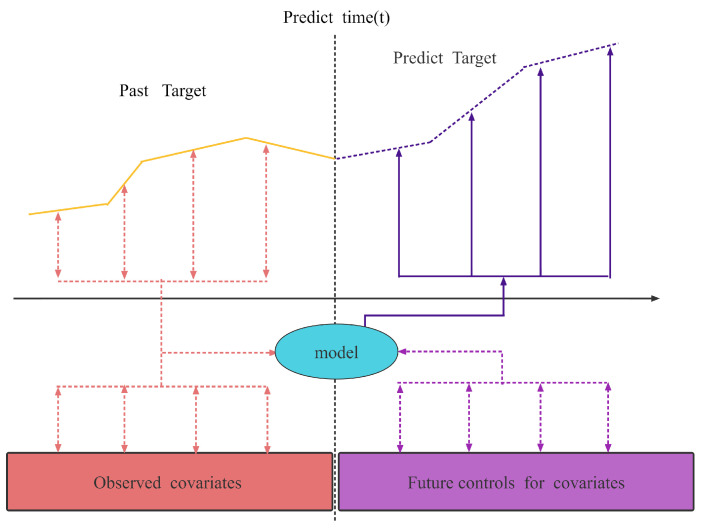
Time series multi-scale variable temperature forecast illustration.

A time series is a sequence of events characterized by continuous-valued variables, and the initial stage of a time series forecasting issue is to determine the target sequence. As shown in Figure 1, the supply air temperature sequence is a prediction sequence in this task. The supply air temperature is the temperature sent into the cabin after the air conditioning cools the air. Different covariates will affect the target series, such as temperature fluctuation, pressure rise, and fall, and compressor start and stop.

In this study, the covariates are divided into two covariates according to the time knowability, as shown in Figure 1: the observed time covariates $x_{i,t}\in R^{m_{x}}$ and the future time control covariates $Z_{i,t}\in R^{m_{z}}$. The observed time covariate is the known sensor observation value. The future time control covariate is the control variable value (compressor frequency, electronic expansion valve opening, and other information) to be predicted within a period. One time step is the data sampling frequency, t∈[0,1,…,n].

There are two forecasting methods for general time series forecasting problems: iterative forecasting and direct forecasting. Each iteration of the iterative prediction method is invoked as the input for the next iterative prediction. The predicted value at time τ is achieved in the iteration τ step. However, the error of the output result is greater, and the efficiency is lower. The direct prediction method outputs the predicted values at t time at one time, with high efficiency and high precision. Therefore, the straightforward prediction method is selected to model and predict the target sequence.

As shown in Figure 1 above, the target sequence, observed covariates, and future control covariates before the current time are used as model inputs to obtain the prediction output of the step. The mathematical model for the sequence prediction problem can be described as:

(1) $${\hat{y}}_{s}\left(t,\tau \right)=f\left(\tau ,y_{t-k:t},z_{i,t-k:t+\tau },x_{i,t-k:t}\right),$$

where ${\hat{y}}_{s}\left(t,\tau \right)$ represents the supply air temperature prediction sequence (i.e., ${\hat{y}}_{s}\left(t,\tau \right)=\left\{{\hat{y}}_{t},{\hat{y}}_{t+1},\ldots ,{\hat{y}}_{t+\tau }\right\}$), τ is the predicted step length, and t is when the prediction starts. Input consists of observed time covariate series (i.e., $x_{i,t-k:t}=x_{t-k},\ldots ,\;x_{t}$) and future time control covariate series (i.e., $z_{i,t-k:t+\tau }=\left\{z_{t-k},\ldots ,z_{t},\ldots ,z_{t+\tau }\right\}$).

### 2.2. Temporal Convolutional Neural Network

Temporal Convolutional Neural Network (TCN) is the causal convolutional network that targets the convolutional structure of time series [22]. A sequence modeling network is any function f:X→Y that produces the mapping. It is taken from supervised learning:

(2) $${\hat{y}}_{t},\ldots ,{\hat{y}}_{t+\tau }=f\left(X_{t-k},\ldots ,\;X_{t+\tau }\right)$$

A given input sequence (i.e., $\left\{X_{t-k},\ldots ,\;X_{t+\tau }\right\}$) corresponding to a given output sequence (i.e., $\left\{{\hat{y}}_{t},{\hat{\;y}}_{t+1},\;\ldots ,\;\;{\hat{y}}_{t+\tau }\right\}$) in ${\hat{y}}_{t}$ can only be affected by the input variables before time t, not by any future input variables; this constraint is a causal constraint. The network produces an output of the same length as the input and the fact that there can be no leakage from the future into the past.

**Figure 2:**
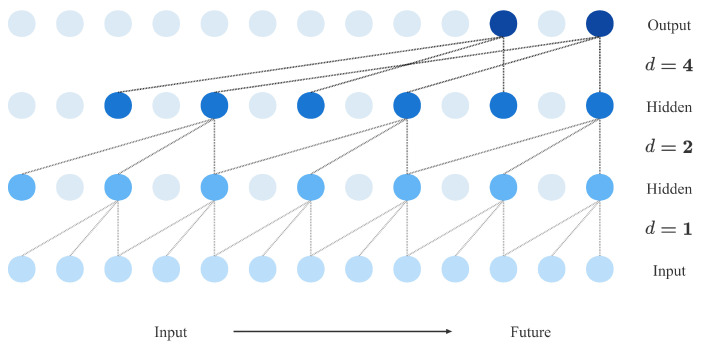
Illustration of an expanded causal convolution with expansion factors.

To solve the causal constraints and increase the receptive field of the convolution kernel, TCN introduces causal convolution with dilated convolution to ensure that future input information will not be known. The dilated convolution operation for a one-dimensional sequence $x\in R^{n}$ of elements s can be described as:

(3) $$F\left(i\right)=\sum_{j=0}^{k-1}h\left(j\right)x_{i-dj}$$

where $F\left(i\right)$ is the convolution result of the *i* element of the sequence *s*, *k* is the filter size (convolution kernel size), *d* is the expansion factor, and * is the convolution operator. Figure 2 shows an example of a dilated causal convolution with dilation factors where dilation factors d=1,2,4 and kernel size k=3. Using larger dilation enables an output at the top level to represent a wider range of inputs, thus effectively expanding the receptive field of a convolution. This gives us two ways to increase the receptive field of the TCN: choosing larger filter sizes *k* and increasing the dilation factor k, where the effective history of one such layer is (k−1)d. As is common when using dilated convolutions, receptive fields increase with the depth of the network. This ensures that some filter hits each input within the effective history while allowing for an extremely effective history using deep networks.

A residual block contains a branch leading out to a series of transformations F, whose outputs are added to the input x of the block:

(4) output=Activation(x+F(x))

**Figure 3:**
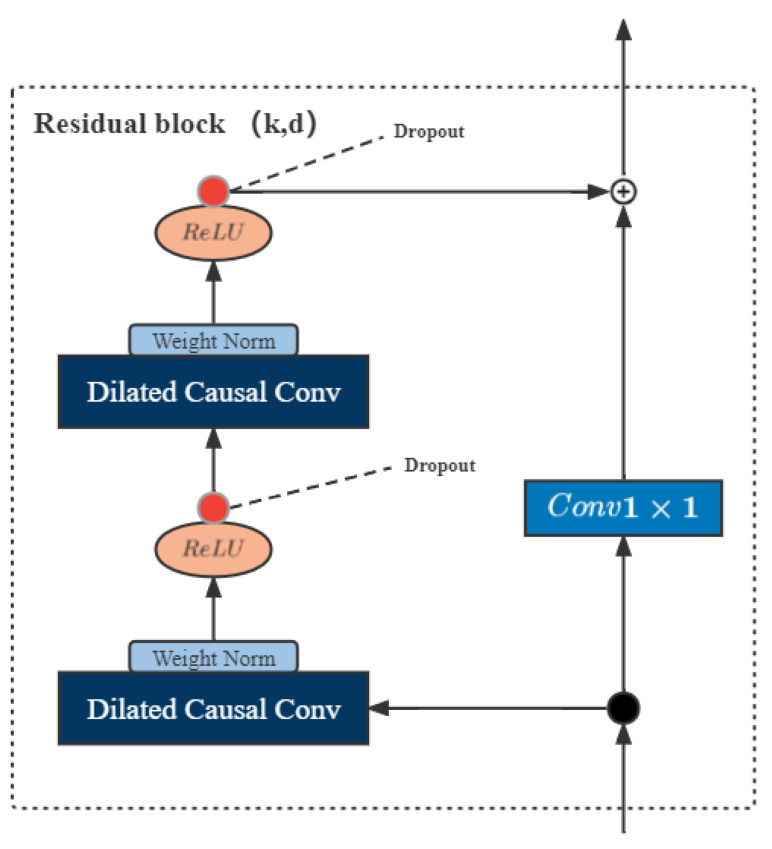
Network structure of TCN.

This effectively allows layers to learn modifications to the identity mapping rather than the entire transformation, which has repeatedly been shown to benefit very deep networks. TCN consists of a one-dimensional fully convolutional network and residual blocks, and residual learning can fully train deep networks. The residual block used in this paper is shown in Figure 3. The residual block is composed of dilated convolution and nonlinear layers. A linear rectification function (ReLU) is used as the activation function. Batch normalization is used for the convolution layer, and unexpected loss is at the end of the convolution. After that, regularization is performed. Finally, the corresponding element-wise summation is performed with the result of the additional 1×1 convolutional layer.

### 2.3. Interpretable Multi-Head Attention

The TFT employs a self-attention mechanism to learn long-term relationships across different time steps and modify from multi-head attention in transformer-based architectures to enhance explainability. In general, attention mechanisms scale values $V\in R^{N\times d_{V}}$ based on relationships between keys $K\in R^{N\times d_{attn}}$ and queries $Q\in R^{N\times d_{attn}}$ as below:

(5) Attention(Q,K,V)=A(Q,K)V

where *N* is the number of time steps inputted to the attention layer, and A() is a normalization function. For attention values, the scaled dot-product is commonly given as follows:

(6) $$A\left(Q,K\right)=\mathrm{Softmax}\left(\frac{QK^{T}}{\sqrt{d_{attn}}}\right)$$

The multi-attention mechanism can learn multiple long-term dependencies at different times. For the learning capacity of the attention mechanism, multi-head attention is adopted to employ other heads for different representation subspaces:

(7) $$H_{h}=\mathrm{Attention}\left(QW_{Q}^{\left(h\right)},KW_{K}^{\left(h\right)},VW_{V}^{\left(h\right)}\right)$$

(8) $$\;\mathrm{MultiHead}\;\left(Q,K,V\right)=\left[H_{1},\ldots ,H_{m_{H}}\right]W_{H}$$

where $W_{Q}^{\left(h\right)}\in R^{d_{\mathrm{model}\;}\times d_{\mathrm{attn}\;}}$ is the weight matrix acting on *Q*. $W_{K}^{\left(h\right)}\in R^{d_{model}\times d_{attn}}$ is the weight matrix acting on *K*, $W_{V}^{\left(h\right)}\in R^{d_{model}\times d_{V}}$ is the weight matrix acting on *V*. *h* is the number of attention heads. $d_{\mathrm{model}\;}$ represents the feature dimension, $d_{\mathrm{attn}\;}=d_{\mathrm{model}\;}/h$. $W_{H}\in R^{\left(h\cdot d_{V}\right)\times d_{\mathrm{model}\;}}$ is the weight matrix of multi-head attention.

Given that each head uses a different value, individual attention weights do not represent the importance of a particular feature. Therefore, multi-head attention is modified to share values in each head, and an additive aggregation of all heads is employed:

(9) $$\tilde{H}=\frac{1}{h}\sum_{1}^{h}\;\mathrm{Attention}\;\left(QW_{Q}^{\left(h\right)},KW_{K}^{\left(h\right)},VW_{V}\right)$$

(10) $$\;\mathrm{InterpretableMultiHead}\;\left(Q,K,V\right)=\tilde{H}W_{H}$$

where $W_{V}\in R^{d_{model}\times d_{V}}$ values weights shared across all heads, and $W_{H}\in R^{d_{attn}\times d_{\mathrm{model}\;}}$ is used for final linear mapping.

### 2.4. Shape and Time Distortion Loss Function

**Figure 4:**
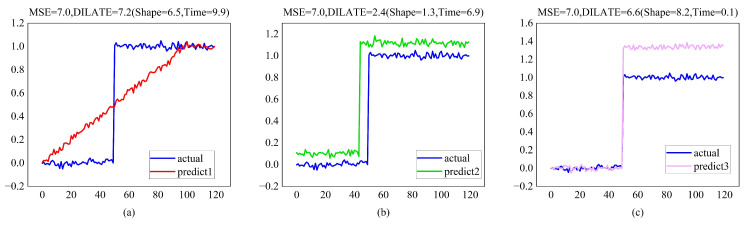
Limitation of the Euclidean (MSE) loss: when expecting a sudden change (target blue step function), predictions (**a**–**c**) have comparable MSE but vastly differing forecasting abilities. In contrast, the DILATE loss disentangles form and temporal decay factors, hence favoring predictions (**b**,**c**) over prediction (**a**), which does not describe the abrupt shift in position.

Regarding training, most methods use the mean squared Error (MSE) or its variants (MAE, etc.) as loss functions. However, relying on MSE in training tasks may not be enough, as shown in Figure 4.

Dynamic Time Warping (DTW) is a method for calculating the similarity between two time series. It dynamically aligns the two sequences by better measuring the two variables’ similarity. Distortion Loss, including Shape and Time(DILATE), is a new objective function for training deep neural networks in multi-step and non-stationary time series forecasting. DILATE explicitly disentangles the penalization related to the shape and the temporal localization errors of change detection:

(11) $$L_{\mathrm{DILATE}\;}\left(x,y\right)=\alpha \cdot L_{\mathrm{shape}\;}\left(x,y\right)+\left(1-\alpha \right)\cdot L_{\mathrm{temporal}\;}\left(x,y\right)$$

where $L_{\mathrm{shape}\;}\left(x,y\right)$ is the shape loss function, and $L_{\mathrm{temporal}\;}\left(x,y\right)$ is the temporal loss function; α∈[0,1] is a hyperparameter that balances the two-loss functions, x (i.e., $x=\left\{x^{1},\ldots ,x^{n}\right\}$) is the target sequence of length *n*, and y (i.e., $y=\left\{y^{1},\ldots ,y^{n}\right\}$) is the predicted sequence of length *n*.

- Shape loss:
  (12) $$L_{\mathrm{shape}\;}\left(x,y\right)=dtw_{\gamma }\left(x,y\right)$$
  (13) $$\begin{array}{cc} dtw_{\gamma }\left(x,y\right) & =min^{\gamma }\left\{\langle A,\Delta \left(x,y\right)\rangle ,A\in A_{n,m}\right\}\\ \; & =-\gamma \log\left(\sum_{A\in A_{n,m}}e^{-\langle A,\Delta (x,y)\rangle /\gamma }\right) \end{array}$$
  (14) $$\Delta \left(x,y\right)={\left[\delta_{i,j}\right]}_{n\times n}\in R^{n\times n}$$
  (15) $$r_{i,j}=\delta_{i,j}+\min^{\gamma }\left\{r_{i,j-1},r_{i-1,j},r_{i-1,j-1}\right\}$$
  (16) $$\min^{\gamma }\left\{a_{1}\ldots a_{n}\right\}=\left\{\begin{array}{cc} \min_{i\leq n}a_{i}, & \gamma =0\\ -\gamma \log\sum_{i=1}^{n}e^{-\frac{a_{i}}{\gamma }}, & \gamma >0 \end{array}\right.$$
  (17) $$r_{n,n}=dtw_{\gamma }\left(x,y\right)$$
  where $A_{n,m}\subset {\left\{0,1\right\}}^{n\times n}$ is the set of calibration matrices for two sequences of length *n*, which represent the range from $\left(x^{1},y^{1}\right)$ to $\left(x^{n},y^{n}\right)$ all paths. A is a path in $A_{n,m}$. Δ(x,y) is the cost matrix composed of two sequences, and $\delta_{i,j}$ is the cost of the corresponding position. Equations (15) and (16) are the “optimal” paths for solving the two sequences, where γ is a hyperparameter, and when it is zero, the solution process is non-differentiable.
- Temporal loss:
  (18) $$L_{\mathrm{temporal}\;}\left(x,y\right)=\langle A^{*},\Omega \rangle =\langle \underset{A\in A_{n,n}}{\mathrm{argmin}}\langle A,\Delta \left(x,y\right)\rangle ,\Omega \rangle$$
  (19) $$\Omega ={\left[w_{i,j}\right]}_{n\times n}\in R^{n\times n}$$
  (20) $$w_{i,j}=\frac{1}{n^{2}}{\left(i-j\right)}^{2}$$
  where Ω is a square matrix of size n×n penalizing each element being associated with an x, for i≠j. $A^{*}$ is the “optimal” path obtained by computing $dtw_{\gamma }\left(x,y\right)$. For $L_{\mathrm{temporal}\;}\left(x,y\right)$, the purpose is to penalize the matching with excessive delay in the DTW algorithm.

## 3. Improved Temporal Fusion Transformers Model

**Figure 5:**
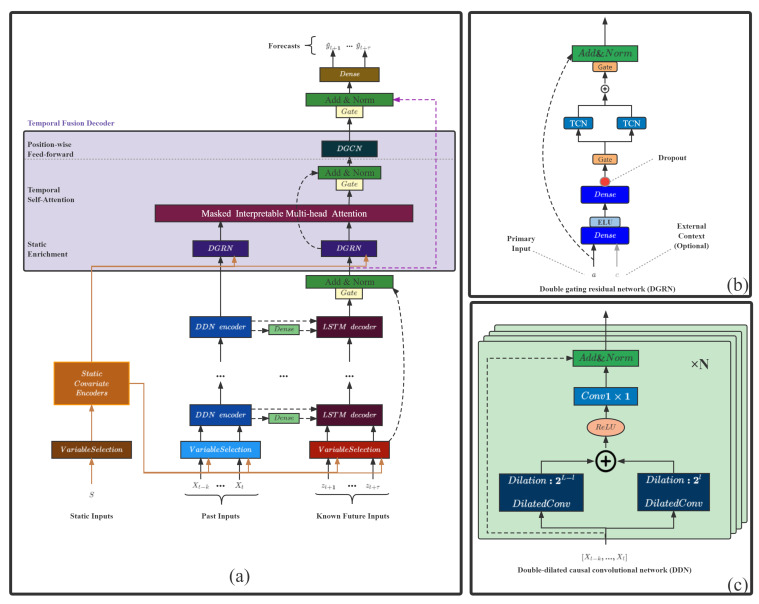
(**a**) Improved TFT architecture. Its inputs include static, time-varying, and time-varying a priori has known future inputs; (**b**) double gating residual network(DGRN). It is a gated residual network with two dimensions of space and time. (**c**) Double-dilated causal convolutional network (DDN) has two causal convolutions with different dilation strides. This means that the receptive fields in both shallow and deep layers can be more flexible.

This study designs a TFT-based temperature prediction framework, as shown in Figure 5a, that uses canonical components to efficiently build feature representations for each input type (i.e., static, known, observed inputs) for high forecasting performance on long-term prediction problems. Two adaptive improvements have been made to the TFT structure, as follows:

- Double-dilated causal convolutional network (DDN): a double-convolutional residual encoder structure based on dilated causal convolution, which has a flexible receptive field; the double-convolutional structure enables shallow layers to capture local and distant information; and its residual structure solves the problem of gradient explosion in deep networks.
- Double gating residual network (DGRN): The spatio-temporal double-gated structure based on Gated Linear Units aims to select items related to the target from the spatial and temporal dimensions and eliminate the influence of noise in the data. The time-gated structure is based on two TCN structures, and the space-gated structure is based on the gated residual network in TFT.

This section first introduces two improved modules and finally introduces the entire model structure.

### 3.1. Double-Dilated Causal Convolutional Network

When dealing with long sequence data, having a more flexible receptive field and capturing more distant information is vital for prediction accuracy. As shown in Figure 5c, the structure of the DDN decoder is a double-dilated causal convolution [23]. One dilated causal convolution raises the dilation factor as the number of layers increases, while the other lowers it as the number of layers increases. The receptive field expands, and localized information can also be perceived in the deep layer when processing time-series data in parallel, convolutional encoder layers with stable gradients, and variable receptive fields have more potential than recurrent neural networks.

For a sequence X (i.e., $X=\left\{x_{1},x_{2},\ldots ,x_{n}\right\}$, $X\in R^{n}$), the convolution output of the $x_{i}$-th element is $F_{l,k}\left(i\right)$ (i.e., $F_{l,k}=\left\{F_{l,k}\left(1\right),F_{l,k}\left(2\right),\ldots ,F_{l,k}\left(n\right)\right\}$). The sequence is first input to the convolution of two dilation factors of different sizes, then the results of the two dilated causal convolutions are added and then activated by the ReLU activation function, and finally a 1×1 convolution layer is added to the input sequence. Each layer’s mathematical description is as follows:

(21) $$F_{l,1}\left(i\right)=\sum_{j=0}^{k-1}h\left(j\right)x_{i-(L-l)j}$$

(22) $$F_{l,2}\left(i\right)=\sum_{j=0}^{k-1}h\left(j\right)x_{i-lj}$$

(23) $${\hat{F}}_{l}=\mathrm{ReLU}\left(\left[F_{l,1}+F_{l,2}\right]\right)$$

(24) $$F_{l}=F_{l-1}+W*{\hat{F}}_{l}+b$$

where *k* is the filter size (convolution kernel size), $W\in R^{n\times n}$ is the weight matrix of the 1×1 convolutional layer, and $b\in R^{n}$ is the bias of the 1×1 convolutional layer.

### 3.2. Double Gating Residual Network

The precise relationship between exogenous inputs and targets is often unknown in advance, making it difficult to anticipate which variables are relevant. However, these effects are not only the unknown adverse effects of different feature variables in space but also the unknown adverse effects of the same feature variables in time. Therefore, a spatial-temporal dual-gated residual network structure is proposed under the original architecture to remove the adverse effects of these features.

As shown in Figure 5b, the DGRN structure adds a time gate structure to the original variable gate structure and selects variables in the time and space dimensions:

(25) $${\mathrm{GRN}}_{\omega }\left(a,c\right)=\;\mathrm{LayerNorm}\;\left(a+{\mathrm{GLU}}_{\omega 1}\left(\eta_{1}\right)\right)$$

(26) $$\eta_{1}=\left[TCN_{1}\left(\eta_{2}\right),TCN_{2}\left(\eta_{2}\right)\right]$$

(27) $$\eta_{2}={\mathrm{GLU}}_{\omega 2}\left(\eta_{3}\right)$$

(28) $$\eta_{3}=W_{1,\omega }\eta_{4}+b_{1,\omega }$$

(29) $$\eta_{4}=\mathrm{ELU}\left(W_{2,\omega }a+W_{3,\omega }c+b_{2,\omega }\right)$$

where ELU is the Exponential Linear Unit activation function [24], $\eta_{1}\in R^{2m\times n}$ and $\eta_{2},\eta_{3},\eta_{4}\in R^{m\times n}$ are intermediate layers, LayerNorm is standard layer normalization, $\eta_{1}$ is the result of concatenating ${\mathrm{TCN}}_{1}\left(\eta_{2}\right)$ and ${\mathrm{TCN}}_{2}\left(\eta_{2}\right)$, and ω is an index to denote weight sharing.

When $W_{2,\omega }a+W_{3,\omega }c+b_{2,\omega }\gg 0$, the ELU activation would act as an identity function, and when $W_{2,\omega }a+W_{3,\omega }c+b_{2,\omega }\ll 0$, the ELU activation would generate a constant output, resulting in linear layer behavior. TCN is calculated based on Equations (2)–(4), which are calculated in the time dimension.

The GLU is described as follows:

(30) $$\mathrm{GLU}\left(\gamma \right)=\sigma \left(W_{1}\gamma +b_{1}\right)⊙\left(W_{2}\gamma +b_{2}\right)$$

where $\gamma \in R^{n}$ is the input, σ(·) is the sigmoid activation function, $b_{\left(\cdot \right)}\in R^{n}$, and $W_{\left(\cdot \right)}\in R^{n\times n}$ are the biases and weights, n is the hidden state size, and ⊙ is the element-wise Hadamard product. ${\mathrm{GLU}}_{\omega 1}$ and ${\mathrm{GLU}}_{\omega 2}$ are calculated in time and space dimensions, respectively.

### 3.3. Improved TFT Model

Transformer-based models have been widely used in time series forecasting. The prediction model designed in this study is based on the TFT model and can include a variety of fusion inputs. Except for two improved structures, the main improved TFT structures are as follows:

- Variable selection networks:
  The variable selection network based on the GRN gated residual network can offer insights into which variables are the most critical to the prediction problem.
- Static covariate encoders:
  The static covariate encoder network integrates static feature variables into the network, through the encoding of context vectors to condition temporal dynamics.
- Interpretable multi-head attention:
  Interpretable multi-head attention is an interpretable multi-head attention mechanism that learns long-term relationships between different time steps.

## 4. Loss Function

The prediction value of more time steps is usually required to predict supply air temperature. For the temperature prediction of more actions, the error of the prediction value of the farther time step position is more significant. This study uses the DILATE loss function to include a temporal distance penalty term to boost learning of the long-distance step feature.

(31) $$L_{\mathrm{loss}\;}\left(x,y\right)=\alpha \cdot L_{1}\left(x,y\right)+\left(1-\alpha \right)\cdot L_{\mathrm{temporal}\;}\left(x,y\right)$$

(32) $$L_{1}\left(x,y\right)=\beta \cdot L_{\mathrm{long}\;}\left(x,y\right)+\left(1-\beta \right)\cdot L_{\mathrm{shape}\;}\left(x,y\right)$$

(33) $$L_{\mathrm{long}\;}\left(x,y\right)=\langle \underset{A\in A_{n,n}}{\mathrm{argmin}}\langle A,\Delta \left(x,y\right)\rangle ,\bar{\Omega }\rangle$$

(34) $$\bar{\Omega }={\left[{\bar{W}}_{i,j}\right]}_{n\times n}\in R^{n\times n}$$

(35) $${\bar{w}}_{i,j}=\frac{i^{2}+j^{2}}{2n^{2}}$$

where the mathematical representation of $L_{\mathrm{temporal}\;}$ is Equations (18)–(20), and the mathematical representation of $L_{\mathrm{shape}\;}$ is Equations (12)–(17), where $\bar{\Omega }$ is a square matrix of size n×n that amplifies long-distance losses.

## 5. Experimental Results and Discussion

In the case of studies, sensors and operating data of the air-conditioning system in a high-speed train carriage at a particular location in China from 11 to 20 June 2020 are applied to assess the forecasting performance of the proposed Improved TFT model. This section presents the implementation of deep learning models by Python 3.7 with TensorFlow 2.8.0, PyTorch-forecasting 0.9.2, PyTorch-lightning 1.5.10, and PyTorch 1.10.2. The data are separated using the Python module “TimeSeriesDataSet”. The model is trained on the GPU and employs an ADAM optimizer. Early Stopping is a technique for avoiding overfitting. The computation is evaluated on an efficient computer with an Intel (R) Core (TM) i7-10875H CPU, NVIDIA GeForce RTX 2060 GPU, and Windows 10 system.

### 5.1. Data Descriptions and Pre-Processing

The high-speed train air conditioning data for ten days (from 11 to 20 June 2020) are collected from a section of a high-speed railway in Shanghai, China. Shanghai is the economic and financial center of China, and the high-speed rail in Shanghai is the primary travel tool for business people and tourists. Improving air conditioning control and predicting the air temperature of air conditioners in the future is vitally essential for high-speed rail modern technology development and passenger thermal comfort.

**Figure 6:**
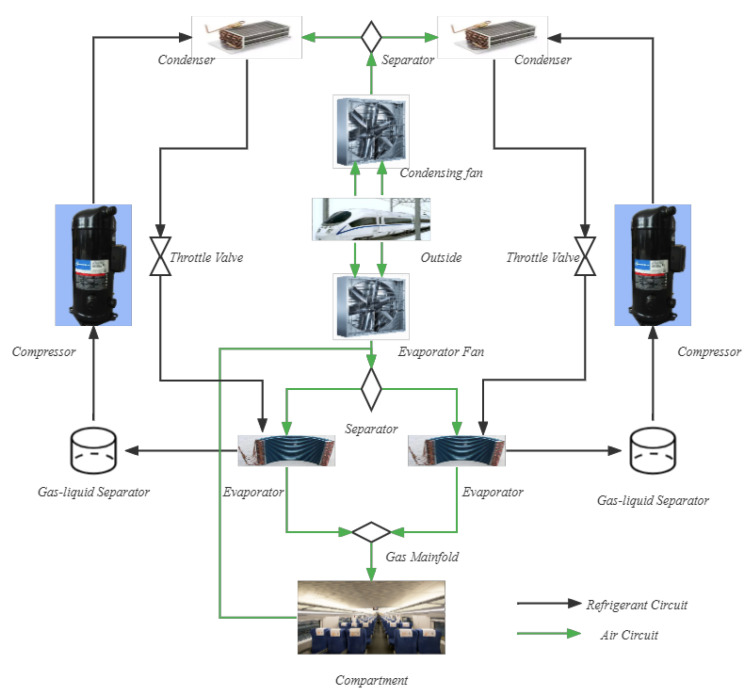
Air conditioning system structure. It includes refrigerant and air circuits. The air cooling process takes place in the evaporator. The gaseous refrigerant becomes a high-pressure gas state after being compressed by the compressor. It is sent to the condenser for heat dissipation to become an average temperature and high-pressure liquid refrigerant. The electronic expansion valve adjusts the refrigerant flow sent to the evaporator, and the pressure of the liquid refrigerant decreases due to the space increase. It vaporizes and dissipates heat to complete the cooling of the air.

In this study, the air-conditioning system includes two identical air-conditioning units installed on the top of the high-speed rail passenger compartment, a ventilation system, and a supporting control system. The dual-compressor parallel inverter air conditioning unit investigated in this work consists of two compressors, two condensers, two electronic expansion valves, two evaporators, two gas–liquid separators, one evaporating fan, and one condensing fan. An air-conditioning unit consists of a refrigerant circuit and an air circuit to form a dynamic flow system, as shown in Figure 6.

**Figure 7:**
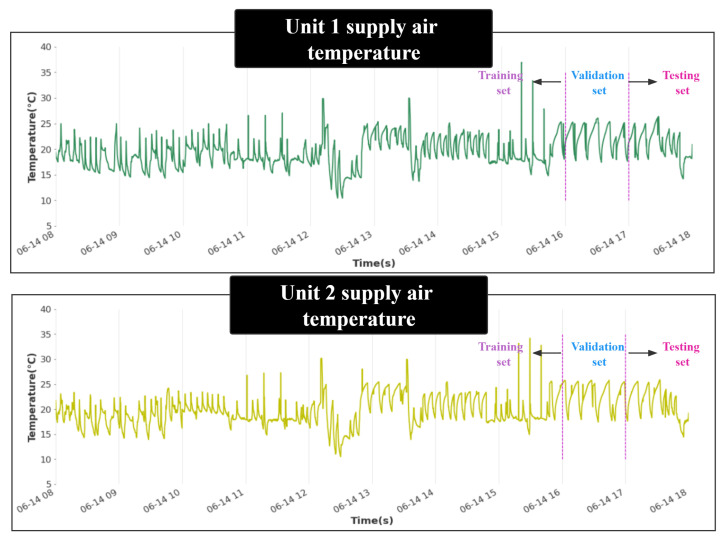
Supply air temperature of unit 1 and unit 2.

This study collected the operation data of the high-speed rail air-conditioning system for ten days (from 8:00 a.m. to 6:00 p.m. every day), with a sampling frequency of 1 s and a total of 360,000 pieces of data. The specific data information is shown in Table 1. In order to improve the generalization ability of the model, the operating data of the air-conditioning system for ten days are divided into days, and 80% of each day is taken as the training set, 10% is the validation set, and 10% is the test set. As shown in Figure 7, the supply air temperature data on June 14 was divided into a training, validation, and test set. The three groups are adopted to build models, select hyperparameters, and verify the final model in sequence.

Table 1 presents the Pearson and Spearman Correlation Coefficient between covariates data and supply air temperature. The Pearson and Spearman test results show that, in the data sets, supply air temperature and suction temperature have a positive correlation, other data have a negative correlation with supply air temperature, and the two units have a robust linear correlation. The outdoor fan frequency has the strongest negative correlation with supply air temperature. In contrast, the suction temperature has the strongest positive correlation, and its correlation to this unit is more significant than that of another unit.

### 5.2. Parameter Setting

The improved model in this study uses an Adam optimizer for optimization training; in addition, the dataset is divided into three parts—a training set for learning, a validation set for hyperparameter tuning, and a test set for performance evaluation. In this study, early stopping was also implemented, and the validation set was utilized to choose the optimum model for training during model training.

The search range of the improved TFT parameters is as follows: prediction time step because the actual demand is 30 steps; DDN Encoder layer, [1, 16]; Number of batch sizes, [16, 512]; State size, [32, 256]; Learning rates, [0.0001, 0.1]; Number of attention heads, [1, 8]; Dropout rate, [0, 0.4]; Loss Function α, [0.1–0.9]; Loss Function β, [0.0001, 0.5]; Loss Function γ, [0.1, 0.5]. The final parameters of the improved TFT of the two units are shown in Table 2.

### 5.3. Evaluation Metrics

Three scale-dependent errors and two percentage errors, namely, MAE, MSE, RMSE, MAPE, and SMAPE, are utilized to evaluate forecasting performance. MAE (Mean Absolute Error) is the average value of the absolute error, which can better reflect the actual situation of the predicted value error. RMSE (Root Mean Square Error) measures the deviation between the observed and actual values. MSE (Mean Square Error) is the square of the difference between the actual value and the predicted value and then summed and averaged. MAPE (Mean Absolute Percentage Error), range [0,+∞), 0% means a perfect model, and 100% means an inferior model. SMAPE (Symmetric Mean Absolute Percentage Error) is a correction index for the problem of MAPE, which can better avoid the problem that the calculation result of MAPE is too large because the real value is small. Equations (36)–(40) give the calculation of the five evaluation metrics:

(36) $$MAE=\frac{1}{\tau }\sum_{t=1}^{\tau }\left|\left({\hat{y}}_{t}-y_{t}\right)\right|$$

(37) $$MSE=\frac{1}{\tau }\sum_{t=1}^{\tau }{\left({\hat{y}}_{t}-y_{t}\right)}^{2}$$

(38) $$RMSE=\sqrt{\frac{\sum_{t=1}^{\tau }{\left({\hat{y}}_{t}-y_{t}\right)}^{2}}{\tau }}$$

(39) $$MAPE=\frac{\sum_{t=1}^{\tau }\left|{\hat{y}}_{t}-y_{t}\right|/y_{t}}{\tau }\times 100\%$$

(40) $$SMAPE=\frac{1}{\tau }\sum_{t=1}^{\tau }\frac{\left|{\hat{y}}_{t}-y_{t}\right|}{\left(\left|{\hat{y}}_{t}\right|+\left|y_{t}\right|\right)/2}$$

τ is the size of output samples, $y_{t}$ is the actual value, and $\hat{y_{t}}$ is the forecasting value. The improvement rate (IR) is introduced to compare the forecasting performance of two different models. The five improved percentage metrics are calculated as follows:

(41) $$IR_{MAE}=\frac{MAE_{A}-MAE_{B}}{MAE_{B}}\times 100\%$$

(42) $$IR_{MSE}=\frac{MSE_{A}-MSE_{B}}{MSE_{B}}\times 100\%$$

(43) $$IR_{RMSE}=\frac{RMSE_{A}-RMSE_{B}}{RMSE_{B}}\times 100\%$$

(44) $$IR_{MAPE}=\frac{MAPE_{A}-MAPE_{B}}{MAPE_{B}}\times 100\%$$

(45) $$IR_{SMAPE}=\frac{SMAPE_{A}-SMAPE_{B}}{SMAPE_{B}}\times 100\%$$

where $IR_{MAE}$, $IR_{MSE}$, $IR_{RMSE}$, $IR_{MAPE}$, and $IR_{SMAPE}$ represent the IRs of model A compared with model B in terms of MAE, MSE, RMSE, MAPE, and SMAPE, respectively.

### 5.4. Ablation Analysis

To validate and quantify the benefits of the two improvements proposed in this study, an extensive ablation analysis was performed removing each component from the network as shown below and quantifying its training results using $IR_{MAE}$, $IR_{MSE}$, $IR_{RMSE}$, $IR_{MAPE}$, and $IR_{SMAPE}$:

(1) DDN encoder layer: This study ablates by replacing the LSTM Encoder with the DDN Encoder designed for this study. The DDN Encoder (Equations (21)–(24)) has a flexible receptive field, which can consider both local and long-distance information.
(2) DGRN layer: The spatiotemporal double-gated (Equations (25)–(30)) structure filters the signal or noise that harms the target in two dimensions, adding only a time-gated unit to the original GRN.

**Figure 8:**
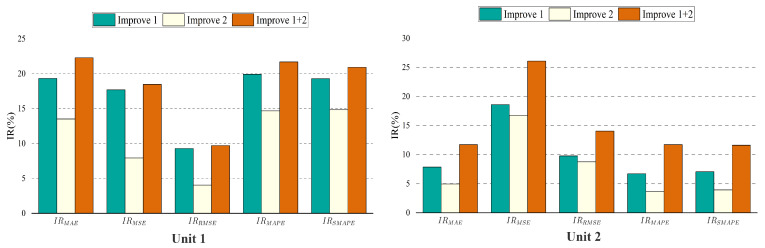
Results of ablation analysis. Improve1 is to add a DDN encoder layer to replace the LSTM encoder layer on the TFT model; improve2 is to add a DGRN layer to the TFT model to replace the GRN layer. IR is the boost ratio compared to the TFT model, Equations (41)–(45).

The ablation network is trained on the dataset using the same hyperparameters. Figure 8 shows that the effects of losses on unit 1 and unit 2 are similar, and all improvements are beneficial to the overall performance.

The TCN structure is generally more effective than the RNN structure in dealing with long-term series prediction problems. The experimental results (MAPE) also show that the DDN Encoder layer with the TCN structure performs better, which is 19.93% and 6.70% higher than the original method in the temperature prediction of unit 1 and unit 2, respectively.

Furthermore, there are not many parameters to tune for the training of the improved network.

The DDN Encoder structure, which has a more flexible receptive field and is better at capturing long-distance features, is more suitable for supply air temperature prediction tasks than the LSTM Encoder structure. The ablation experiment results of the DGRN structure show that the temperature prediction results (MAPE) of unit 1 and unit 2 are improved by 14.68% and 3.64%, respectively, compared with the original model. The ablation experiments’ results show that enhancing the gating structure improves the model efficiency and successfully filters the values that negatively affect future temperature predictions in the time dimension.

**Figure 9:**
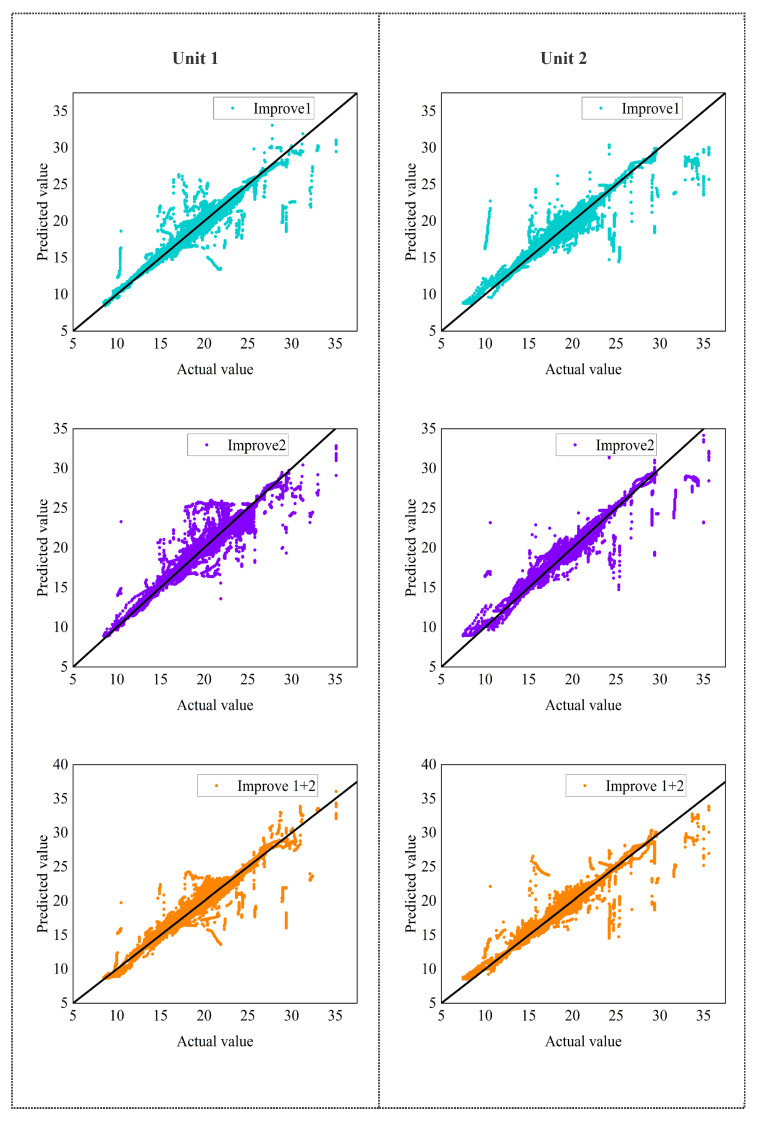
Ablation experiment correlation plot.

In addition, through the analysis of Figure 9, when improvements one and two are used in the prediction model simultaneously, they will play a mutually reinforcing role. Moreover, when different improvements have significant differences in prediction performance at the exact location, the improvement with better performance will dominate the prediction at this location.

When the last two improvements are applied to the prediction model simultaneously, there is no conflicting problem, and all five evaluation indicators are improved. In particular, the prediction of the supply air temperature of unit 1 is significantly enhanced (the MAPE loss is increased by 21.70%), indicating that it is more effective in noisy data.

### 5.5. Results and Discussion

The prediction results of Improved-TFT on the high-speed rail Air Conditioning dataset compare with those of other methods regarding MAE, MSE, RMSE, MAPE, and SMAPE. Table 3 presents the forecasting performance of each model. The results are analysed in detail below.

This study selected seven algorithms for comparison experiments: random forest prediction (RF), LSTM, GRU [25], TCN, Transfomer [26], N-BEATS [27], and N-HITS [28]. These methods represent typical machine learning, RNN networks, CNN networks, Transformers, and several state-of-the-art multivariate long-term prediction methods. These models represent the most popular forecasting methods, with random forests being the first of the seven to be proposed and widely used over a while.

**Figure 10:**
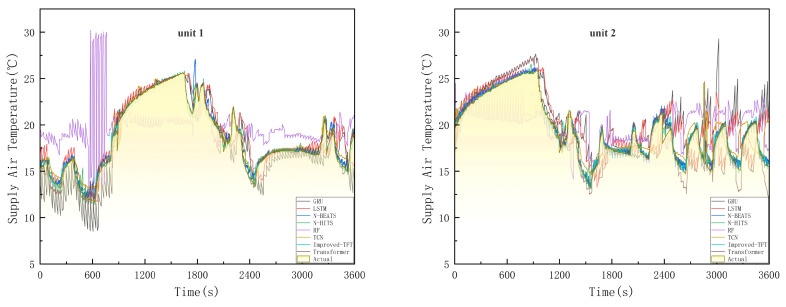
Experimental results of air-conditioning supply air temperature prediction for different models.

Table 3 and Figure 10 show that the improved TFT model significantly outperforms the tests of all comparison methods. The MAPE values of the Unit 1 prediction model: random forest prediction, LSTM, GRU, TCN, Transfomer, N-BEATS, N-HITS, and improved TFT are 8.64%, 13.00%, 13.10%, 3.19%, 1.93%, 2.10%, 2.12%, and 1.20%, respectively. The MAPE values of the Unit 1 prediction model: random forest prediction, LSTM, GRU, TCN, Transfomer, N-BEATS, N-HITS, and improved TFT are 8.71%, 15.06%, 15.03%, 3.34%, 2.53%, 2.33%, 2.35%, and 1.30%, respectively. Compared with the sub-optimal model, the improved TFT model significantly improves the air-conditioning temperature prediction task. The MAPE of unit 1 is reduced by 0.71%, and the MAPE of unit 2 is reduced by 1.02%. The results reveal that the improved TFT model is the best individual prediction model.

An improved TFT model with multiple variables can gain more information in prediction problems, and its future known inputs contain a lot of information that positively affects the prediction results. The attention mechanism shows excellent performance in the experiments, and the performance becomes even better after the improved TFT model aligns these inputs. Moreover, the experimental results also show that the prediction model of the CNN structure is far worse than the prediction model of the TCN structure in the supply air temperature prediction, which confirms that it is unsuitable for a long-time prediction task discussed.

### 5.6. Interpretability Analysis

**Figure 11:**
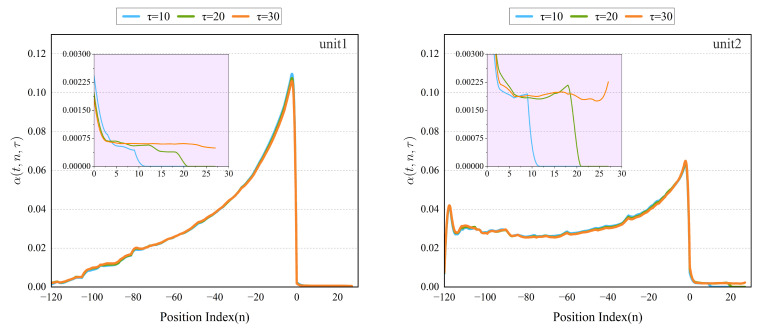
Interpretability experimental results for improved TFT.

This section clarifies the interpretability of the improved TFT model, analyzing its relationship with time. As shown in Figure 11, in the past time information of unit 1, the data close to the predicted time shows a strong correlation because the more immediate information has a more significant impact on the prediction of the supply air temperature. The farther position data may be subject to substantial environmental changes due to the high-speed rail.

Through the attention analysis of unit 1 and unit 2, the attention weight of the past time shows an overall upward trend with the positive sequence of time; the attention weight of the future time has different degrees of hysteresis, and the overall is first decreased, then slowly increased, and finally has a further downward trend. Experts researching and developing air conditioners agree with such a hysteresis result. Attentional weight patterns can elucidate the model’s most important past time steps based on its decisions. Compared to traditional and machine learning time series methods, which rely on model-based norms for lag analysis, the improved TFT can learn this pattern from raw training data.

In the future information of unit 1, the data close to the prediction time shows a weak correlation because the prediction model has a certain lag. As shown in Figure 11, the overall importance of the past information in unit 2 is similar to that in unit 1. However, its volume fluctuates to a certain extent at positions farther from the predicted point. The difference in the importance of future information for unit 2 and unit 1 is that it has increased in recent times. The interpretability experiments of unit 1 and unit 2 show that the temporal dynamic trends of the two units are roughly the same, but there are also some differences. A possible explanation is that this difference occurs because the control algorithm of the high-speed rail air conditioner controls the two units. In the operation process of the two units, only one is often running.

## 6. Conclusions

Air conditioning control mainly changes the supply air temperature. However, various factors such as the ambient temperature, the speed of the high-speed rail, and the high and low pressure of the air conditioning system will also affect its changes. With the popularity of high-speed rail in China, passengers who choose to travel by high-speed rail are more and more concerned about the ride’s comfort. Different from the environment of ordinary air conditioners, the environment of high-speed rail changes very fast, and the air temperature of air conditioners fluctuates wildly, which not only leads to a decrease in thermal comfort but also increases the energy consumption of air conditioners. Accurate prediction is a meaningful job.

This study introduces a unique interpretable predictive model, TFT, with specific improvements. Extensive experiments have proved that the TCN structure is suitable for time series processing, so this study selects TCN to improve the TFT model. The results of ablation experiments demonstrate that the improvements discussed in this study are practical. Experiments show that, compared with seven popular models, the improved TFT model has the best performance in predicting supply air temperature, among which the experimental results of unit 1 have the best experimental results compared with the seven models in MAE, MSE, RMSE, MAPE, and SMAPE improved by 0.13, 0.47, 0.31, 0.71%, and 0.72%. Finally, in terms of the interpretability of the model, only the interpretable analysis of the time dimension is done, and the interpretability of the entire prediction problem is the main work to be done in the future. For the air conditioner temperature prediction task, the attention weight of this study has been recognized by experts, and an objective fact has been learned through the training of the model, that is, the change of the external environment or the rise and fall of the compressor frequency cannot immediately change the air conditioner temperature. The study of interpretability is critical if engineering applications want to earn the trust of human experts in the future.

## Figures and Tables

**Table 1:** Covariate correlation coefficients table (Part).

	Covariates	Unit 1 Supply Air Temperature		Unit 2 Supply Air Temperature	
		Pearson	Spearman	Pearson	Spearman
Unit 1	Condensing Inlet Air Temperature	−0.466	−0.478	−0.493	−0.518
	Outdoor fan frequency	−0.527	−0.568	−0.329	−0.369
	Compressor 1 inverter frequency	−0.438	−0.420	−0.282	−0.267
	Compressor 2 inverter frequency	−0.427	−0.395	−0.297	−0.294
	Electronic expansion valve 1	−0.337	−0.333	−0.265	−0.225
	Electronic expansion valve 2	−0.392	−0.411	−0.335	−0.341
	Suction temperature 1	0.523	0.538	0.458	0.457
	Suction temperature 2	0.466	0.504	0.432	0.475
Unit 2	Condensing Inlet Air Temperature	−0.463	−0.490	−0.470	−0.510
	Outdoor fan frequency	−0.426	−0.452	−0.566	−0.593
	Compressor 1 inverter frequency	−0.317	−0.295	−0.454	−0.417
	Compressor 2 inverter frequency	−0.344	−0.331	−0.444	−0.408
	Electronic expansion valve 1	−0.186	−0.099	−0.221	−0.197
	Electronic expansion valve 2	−0.316	−0.294	−0.366	−0.363
	Suction temperature 1	0.389	0.383	0.507	0.559
	Suction temperature 2	0.451	0.447	0.548	0.569

**Table 2:** Parameters of the Improved-TFT in the high-speed rail air conditioning dataset.

Parameter	Unit 1	Unit 2
Number of time steps	30	30
Number of DDN encoder layers	4	4
Number of batch sizes	64	256
State size	64	64
Learning rates	0.01	0.01
Number of attention heads	4	4
Dropout rate	0.2	0.2
Loss Function α	0.8	0.9
Loss Function β	0.01	0.01
Loss Function γ	0.1	0.05

**Table 3:** Forecasting results of different models in the high-speed air conditioning supply air temperature data set.

Model	Unit 1					Unit 2				
	MAE	MSE	RMSE	MAPE	SMAPE	MAE	MSE	RMSE	MAPE	SMAPE
RF	1.54	5.42	2.33	8.64%	8.18%	1.51	5.73	2.39	8.71%	8.07%
LSTM	2.33	11.48	3.39	13.00%	12.31%	2.66	15.34	3.92	15.06%	13.87%
GRU	2.38	11.52	3.39	13.10%	12.89%	2.68	15.29	3.91	15.03%	14.21%
TCN	0.59	1.32	1.15	3.19%	3.18%	0.62	1.50	1.23	3.34%	3.31%
Transformer	0.36	0.84	0.91	1.91%	1.93%	0.48	1.42	1.19	2.53%	2.57%
N-BEATS	0.39	0.93	0.97	2.09%	2.10%	0.45	1.35	1.16	2.33%	2.35%
N-HITS	0.39	0.96	0.98	2.11%	2.12%	0.43	1.36	1.17	2.35%	2.33%
**OURS**	**0.23**	**0.37**	**0.60**	**1.20%**	**1.21%**	**0.24**	**0.48**	**0.69**	**1.31%**	**1.30%**

## Data Availability

The data are available from the corresponding author of this paper. Data sharing not applicable.
